# Retraction Note: Associations of systemic oxygen consumption with age and body temperature under general anesthesia: retrospective cohort study

**DOI:** 10.1186/s12871-023-02323-6

**Published:** 2023-10-31

**Authors:** Satoshi Kimura, Kazuyoshi Shimizu, Hiroshi Morimatsu

**Affiliations:** Department of Anesthesiology and Resuscitology, Okayama University Hospital, 2-5-1, Shikata-Cho, Kita-Ku, Okayama, 700-8558 Japan

**Retraction Note: BMC Anesthesiology (2023) 23:216**

10.1186/s12871-023-02182-1

The authors have retracted this article. After publication the authors realised that there was an error in the unit of oxygen consumption, the unit was listed as ml/kg/min, instead of 10 − 1 ml/kg/min. As this error affects all of the results and conclusions presented, the authors decided to retract the article. All authors agree to this retraction.

